# Higher Plant Diversity Does Not Moderate the Influence of Changing Rainfall Regimes on Plant–Soil Feedback of a Semi‐Arid Grassland

**DOI:** 10.1111/gcb.70084

**Published:** 2025-03-04

**Authors:** Xiliang Li, G. Kenny Png, Zhen Zhang, Fenghui Guo, Yuanheng Li, Fang Li, Shan Luo, Nicholas J. Ostle, John N. Quinton, Urs A. Schaffner, Xiangyang Hou, David A. Wardle, Richard D. Bardgett

**Affiliations:** ^1^ Institute of Grassland Research Chinese Academy of Agricultural Sciences Hohhot China; ^2^ Department of Earth and Environmental Sciences The University of Manchester Manchester UK; ^3^ The Industrial Crop Institute Shanxi Agriculture University Taiyuan China; ^4^ Lancaster Environment Centre Lancaster University Lancaster UK; ^5^ Department of Evolution, Ecology and Behaviour University of Liverpool Liverpool UK; ^6^ Centre for Agriculture and Biosciences International Delémont Switzerland; ^7^ College of Grassland Science Shanxi Agriculture University Taigu China; ^8^ Department of Ecology and Environmental Science Umeå Universitet Umeå Sweden

**Keywords:** climate change, drought, plant functional groups, plant–soil (below‐ground) interactions, plant–soil feedback, species richness

## Abstract

Climate change is expected to increase the frequency of severe droughts, but it remains unclear whether soil biotic conditioning by plant communities with varying species richness or functional group diversity moderate plant–soil feedback (PSF)—an important ecosystem process driving plant community dynamics—under altered rainfall regimes. We conducted a two‐phase PSF experiment to test how plant diversity affects biotic PSF under different rainfall regimes. In Phase 1, we set up mesocosms with 15 plant assemblages composed of two grasses, two forbs and two nitrogen‐fixing legumes [one, two, three, or six species from one, two, or three functional group(s)] common to the semi‐arid eastern Eurasian Steppe. Mesocosms were subjected to two rainfall amounts (ambient, 50% reduction) crossed with two frequencies (ambient, 50% reduction) for a growing season (~3 months). Conditioned soil from each mesocosm was then used in Phase 2 to inoculate (7% v/v) sterilised mesocosms planted with the same species as in Phase 1 and grown for 8 weeks. Simultaneously, the same plant assemblages were grown in sterilised soil to calculate PSF based on plant biomass measured at the end of Phase 2. Feedback effects differed amongst plant assemblages, but were not significantly altered by reduced rainfall treatments within any plant assemblage. This suggests that the examined interactions between plant and soil microbial communities were resistant to simulated rainfall reductions and that increasing plant diversity did not moderate PSF under altered rainfall regimes. Moreover, increasing plant species richness or functional group diversity did not lessen the magnitude of PSF differences between ambient and reduced rainfall treatments. Collectively, these findings advance our understanding of plant diversity's potential to mitigate climate change effects on PSF, showing that in semi‐arid grasslands, higher plant diversity may not moderate PSF responses to altered rainfall regimes and highlighting the importance of considering species‐specific traits and interaction stability.

## Graphical abstract

Our study tested whether increasing plant diversity helps mitigate the effects of drought on plant‐soil microbial community interactions in a semi‐arid grassland. Using a two‐phase plant‐soil feedback experiment, we found that plant diversity did not significantly alter plant‐soil feedback responses under different rainfall conditions. These findings suggest that plant‐soil interactions of this semi‐arid grassland, including both plant and soil microbial community dynamics, are largely resistant to changes in rainfall, highlighting the need to consider species‐specific traits and interaction stability rather than diversity alone when predicting ecosystem responses to drought.

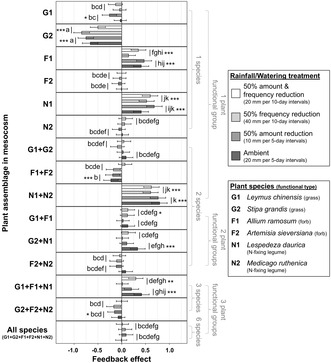

## 1 Introduction

Plant species possess an ability to shape soil biotic and abiotic properties, subsequently affecting the performance of the same or different plant species via plant–soil feedback (PSF) (Bever et al. 1997; van der Putten et al. 2013). Negative feedback occurs if a plant performs worse in soil conditioned by conspecific species when compared with soil conditioned by heterospecific species, whilst the reverse results in positive feedback. There is increasing evidence that PSF can be an important driver of plant community dynamics, and that the direction and strength of these feedback effects are dependent on both plant functional type and environmental factors that can be modified by climate change (Cortois et al. 2016; Fry et al. 2018; Lekberg et al. 2018; Pugnaire et al. 2019; Smith‐Ramesh and Reynolds 2017). With climate change expected to increase the frequency and intensity of anomalous weather events globally (Bardgett et al. 2021; Gang et al. 2014; Pugnaire et al. 2019), understanding how climatic factors influence PSF becomes increasingly crucial. Drought, characterised by lower‐than‐normal precipitation amounts and/or longer dry periods for a specific region and season (Intergovernmental Panel on Climate Change 2023), is one such factor that has been shown to alter the magnitude and direction of PSF effects in several recent studies (Crawford and Hawkes 2020; Dudenhöffer et al. 2022; Fry et al. 2018; Hassan et al. 2021; Kaisermann et al. 2017; Lozano et al. 2022; Snyder and Harmon‐Threatt 2019; Wilschut and van Kleunen 2021), albeit with largely unknown consequences for plant community dynamics (Pugnaire et al. 2019; van der Putten et al. 2016). Moreover, biodiversity, especially plant diversity, is increasingly proposed as a solution to mitigate the negative effects of global change drivers, including drought, on ecosystems and their functioning (Hisano et al. 2018; Isbell et al. 2015; Mori et al. 2021). However, little is known about how plant species richness and functional group diversity mediate PSF responses to changes in rainfall regimes. A better understanding of how plant diversity mediates PSF responses to changes in rainfall regimes could help predict and manage the long‐term consequences of drought on the vegetation composition and functioning of ecosystems under climate change.

Drought effects on PSF responses can occur via various pathways, such as impacting plant physiological responses and their subsequent soil conditioning, and/or direct effects on the abundance and community composition of soil microorganisms (Pugnaire et al. 2019). Prolonged water stress affects plant performance and alters the quantity and quality of plant‐derived inputs (e.g., leaf litter, rhizodeposition and root exudates) into the soil and the rhizosphere (e.g., de Vries et al. 2023; Preece et al. 2019). This, in turn, could influence the composition of beneficial soil microorganisms or species‐specific soilborne pathogens in the rhizosphere, leading to shifts in PSF (Pugnaire et al. 2019). Further, reduced precipitation may also alter PSF effects directly by reducing the abundance and composition of below‐ground biota by favouring certain microbial taxa more than others (e.g., fungi vs. bacteria; Preece et al. 2019; Pugnaire et al. 2019; Thakur et al. 2015). However, it is difficult to generalise how shifts in PSF effects are influenced by reduced water availability because experimental studies are limited (e.g., De Long et al. 2023; Hassan et al. 2022), and findings of existing studies are varied with reports of positive, neutral, or negative shifts in feedback effects during drought (e.g., Dudenhöffer et al. 2022; Fry et al. 2018; Hassan et al. 2021; Lozano et al. 2022; Snyder and Harmon‐Threatt 2019).

Plant functional traits or groups have often been cited as useful predictors of PSF (Baxendale et al. 2014; Cortois et al. 2016; Lozano et al. 2022; Semchenko et al. 2018; Spitzer et al. 2022), as well as of drought tolerance (Mackie et al. 2019; Martorell et al. 2021). A large proportion of previous PSF studies revealed that nitrogen (N)‐fixing plants were often associated with positive feedback effects due to their soil N inputs and promotion of beneficial microorganisms in N‐poor soil, whilst non‐N‐fixing plants such as grasses and other forbs often showed negative feedback effects (Cortois et al. 2016; Hassan et al. 2022; Png et al. 2019). Similarly, broad hydraulic trait differences between contrasting plant functional groups (e.g., generally greater turgor loss point values and greater drought tolerance of forbs compared with graminoids; Májeková et al. 2021; Sun et al. 2020) may also explain plant performance during drought stress which, in turn, affects their strength of influence on soil biotic and abiotic properties. As such, plant functional traits or groups could help explain the maintenance or shift in the direction and strength of PSF when subjected to contrasting rainfall regimes. However, predictions of the direction and magnitude of PSF based on plant functional groups or traits remain difficult, because evidence from the limited number of available studies remains inconsistent (Enderle et al. 2024; Gundale and Kardol 2021; Hassan et al. 2022; Spitzer et al. 2022).

Plant species richness has been shown to increase the resistance of plant community productivity to drought (e.g., Bazzichetto et al. 2024; Isbell et al. 2015; Liu, Wang, et al. 2022), and this could be driven by the nature of interactions of plant roots from different species with each other and their microbial communities (Allison and Martiny 2008; Bardgett and Caruso 2020). For example, N‐fixing plants can accumulate *Rhizobia*—a group of plant‐growth‐promoting bacteria that releases phytohormones that enhance drought tolerance of non‐N‐fixing plants (García‐Fraile et al. 2012; Yong et al. 2014) or elevate soil N availability via symbiotic N fixation (von Gillhaussen et al. 2014; Weidlich et al. 2017) to aid recovery of growth between rainfall events. In addition, plant species with mycorrhizal associations could enhance drought resistance by helping to suppress the effects of soilborne pathogens (Marx 1972; Weng et al. 2022), increasing soil nutrient availability via exudates (Paterson et al. 2016; Plassard et al. 2011), and/or improving soil water availability through enhanced soil structure (Pauwels et al. 2023; Rillig and Mummey 2006) or hydraulic lift (Egerton‐Warburton et al. 2008). Together, interactions amongst a greater diversity of plant species containing such beneficial traits could potentially better support facilitative interactions, which may, in turn, help improve plant community resistance to environmental stressors such as droughts (Isbell et al. 2015; Mariotte et al. 2013; van Ruijven and Berendse 2010). Conversely, increased plant diversity may offer limited benefits (Grime 1998) if the plant community and soil conditioning processes are increasingly dominated by certain competitive, resource‐efficient species under intensifying drought stress (Martínez‐Vilalta and Lloret 2016), and therefore yield feedback effects that are unlike those observed under ambient conditions (Hassan et al. 2022). Nevertheless, little is known about how increasing plant species richness and functional group diversity, and the greater number of facilitative interactions likely to occur in a more diverse plant community, can confer stability on PSF during droughts.

The effect of plant diversity on the resistance of PSF to drought in grasslands, or other terrestrial ecosystems, remains largely unexplored. To address this knowledge gap, we focused on the eastern Eurasian Steppe, one of the world's most expansive grasslands and a major terrestrial ecoregion that provides a wide range of highly valuable ecosystem services such as livestock grazing/production, carbon sequestration, climate regulation and cultural values (Addison and Greiner 2016). Like many grassland ecosystems globally (Bardgett et al. 2021), the semi‐arid steppe is facing increasing pressure from a drier climate (Liu et al. 2013).

To examine whether the strength and direction of biotic PSF effects of a semi‐arid grassland can be modulated by plant species richness or functional group diversity when subjected to contrasting rainfall amounts and frequencies, we conducted a two‐stage intra‐assemblage PSF experiment using common plant species and soil from the eastern Eurasian Steppe, within Inner Mongolia, China. Our focus on intra‐assemblage PSF enabled us to examine how plant diversity within an assemblage influences its constituent species (i.e., the same assemblage) via rainfall‐dependent soil biotic legacy effects. However, it did not allow us to address scenarios influenced by soil conditioning from different plant assemblages, such as those resulting from species replacement or new arrivals. We subjected sheltered outdoor mesocosms containing 15 different planting treatments (15 distinct assemblages drawn from six common plant species from three functional groups—grasses, forbs, or N‐fixing legumes) to either an ambient rainfall regime or regimes with 50% reduction(s) in rainfall amount and/or frequency, and then examined the soil biotic legacy effects on the same plant assemblage (i.e., biotic PSF) in a greenhouse. We hypothesised that (i) reduced rainfall amount or frequency will disrupt plant–soil interactions during the soil conditioning phase sufficiently to alter (or neutralise) the direction and magnitude of biotic PSF effects observed under ambient conditions, and (ii) higher plant species richness and functional group diversity will buffer shifts in the direction and/or magnitude of biotic PSF effects resulting from reduced rainfall amount or frequency.

## 2 Materials and Methods

### 2.1 Study Area

Our study was conducted within a semi‐arid steppe ecosystem of the eastern Eurasian Steppe at the Chinese Academy of Agricultural Sciences‐Institute of Grassland Research (CAAS‐IGR) Shaerqin Research Station (40°34′ N, 111°56′ E), Hohhot, Inner Mongolia Autonomous Region, China. The soil collected and utilised in our study was classified as Castano‐Cinnamon Soils (Chinese soil taxonomy; Shi et al. 2004) or Calcic‐Orthic Aridisol (USDA soil taxonomy; United States Department of Agriculture 2014), with a mean soil organic carbon content of 4.99 g kg^−1^, total N content of 0.62 g kg^−1^, available phosphorus content of 3.43 mg kg^−1^ (Olsen P) and pH of 8.96.

The study area has a cold semi‐arid climate with long, dry and cold winters, but hot, windy summers (China Meteorological Administration [CMA], https://www.cma.gov.cn/). The mean annual temperature is ~7.4°C and the mean monthly temperature ranges between −11.0°C and 23.3°C (1980–2018; CMA, https://www.cma.gov.cn/). The mean annual rainfall is 396 mm, most of which falls between mid‐April and mid‐September (1980–2018; CMA, https://www.cma.gov.cn/).

### 2.2 Plant Species Selection, Seed Collection and Pre‐Treatment

We selected six common species, each widely distributed throughout the Inner Mongolia steppe ecosystems (Li et al. 2020; Liu, Qiao, et al. 2022), to represent three broad plant functional groups: two grasses [(G1) *Leymus chinensis* (Trin.) Tzvelev and (G2) *Stipa grandis* P. A. Smirn.], two (non‐N‐fixing) forbs [(F1) *Allium ramosum* L. and (F2) *Artemisia sieversiana* Ehrh. ex Willd.], and two N‐fixing legumes [(N1) *Lespedeza daurica* (Laxm.) Schindl. and (N2) *Medicago ruthenica* (L.) Trautv.]. *L. chinensis* and *S. grandis* are a rhizomatous and a bunch grass, respectively, and are the most dominant species in Inner Mongolia. The two selected N‐fixing legume species are common perennial species with high nutritional values for livestock grazing. In addition, the other two forbs are the most common forb species that co‐occur in our study area, especially in degraded communities experiencing disturbances such as heavy livestock grazing.

Seeds of the six selected species were collected from a native steppe site in Xilinhot, Inner Mongolia Autonomous Region, China, which is similar to our study area in Hohhot. For each experimental phase, seeds were surface sterilised with a 2% (w/v) sodium hypochlorite solution for 25 min and rinsed three times in sterile deionised water before sowing into the respective Phase 1 mesocosm (cylindrical; diameter = 30 cm, height = 35 cm; free‐draining) during Phase 1, or smaller Phase 2 mesocosms (cylindrical; diameter = 12 cm, height = 16 cm; free‐draining) during Phase 2. To improve the germination rates of the legume species, their seedcoats were abraded with sandpaper to overcome physical dormancy.

### 2.3 Experimental Design

**FIGURE 1:**
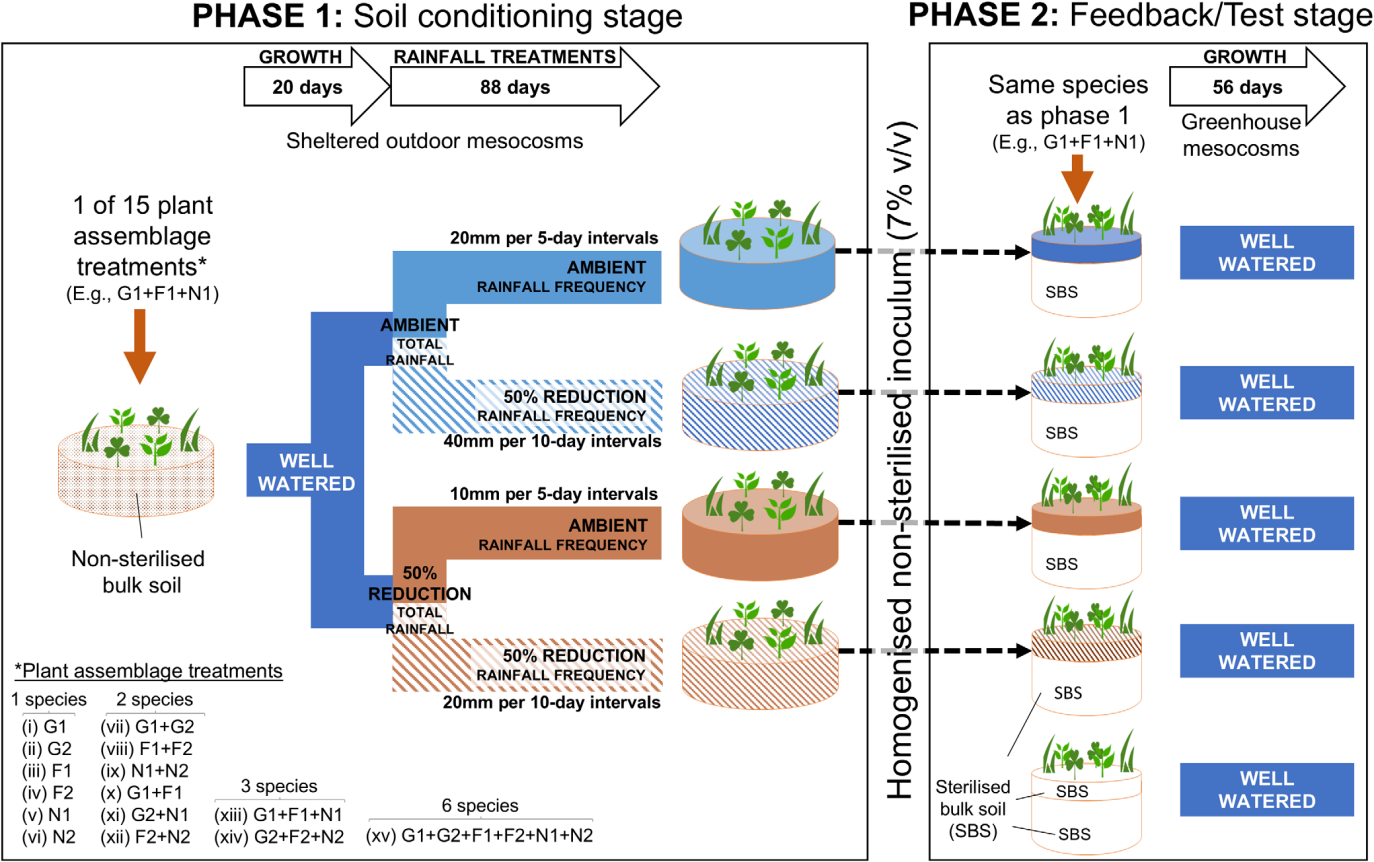
An illustrative example of the experimental setup of one of 15 plant assemblages utilising six common grassland plant species of the eastern Eurasian Steppe to study the influence of plant species and functional group diversity, rainfall amounts and frequencies (ambient and 50% reduction), and their interactions on biotic plant–soil feedback. Treatments illustrated above were replicated four times for every assemblage treatment. G1, *Leymus chinensis* (grass); G2, *Stipa grandis* (grass); F1, *Allium ramosum* (forb); F2, *Artemisia sieversiana* (forb); N1, *Lespedeza daurica* (nitrogen‐fixing legume); N2, *Medicago ruthenica* (nitrogen‐fixing legume).

To assess how biotic PSF involving different levels of plant species richness or functional group diversity may differ under contrasting rainfall amounts and frequencies, we used a two‐phase PSF experiment (Phase 1: conditioning phase, and Phase 2: feedback phase), and measured plant biomass of Phase 2 mesocosms to calculate PSF values (Figure 1). We also employed a ‘non‐sterilised’ versus ‘sterilised’ approach to calculating biotic PSF values (Brinkman et al. 2010; Kardol et al. 2007). In Phase 1, we subjected 15 different plant assemblages in mesocosms to each of two rainfall amounts (ambient or 50% reduction) crossed with two rainfall frequency (ambient or 50% reduction) treatments. We then used soil collected from Phase 1 mesocosms to inoculate (7% v/v) mesocosms in Phase 2 that consisted of a total of 60 treatment combinations, namely: 15 plant assemblages × 2 total rainfall amounts (during Phase 1) × 2 rainfall frequencies (during Phase 1). The 15 plant assemblage treatments used six common plant species to create four levels of plant species richness treatments (1, 2, 3, or 6 species), and three levels of functional group treatments (1, 2, or 3 functional groups).

In Phase 1, we conditioned soils by planting each of the 15 plant assemblages in sheltered outdoor mesocosms containing non‐sterilised bulk soil and then subjected them to rainfall treatments (Figure 1). In Phase 2, each of the 15 plant assemblages was grown in either fully sterilised bulk soils, or sterilised bulk soil inoculated with soil (7% v/v) conditioned by the same Phase 1 plant assemblage previously subjected to the rainfall treatments. We used a completely randomised design, and the experiment was replicated four times for a total of 240 and 300 experimental units for Phases 1 and 2, respectively. Each mesocosm contained six individual seedlings, and mesocosms with a ‘plant assemblage’ treatment requiring two or more species each contained an equal number of seedlings for each required species.

### 2.4 Phase 1: Soil Conditioning, Drought Treatments and Associated Measurements

Phase 1 of the study was conducted using outdoor mesocosms, placed under transparent rain shelters with > 90% light transmittance, at the CAAS‐IGR Shaerqin Research Station for the growing season between June and September 2020. The mean monthly temperature between June and September 2020 ranged from 10.0°C to 29.5°C, and this period generally receives the most rainfall annually (CMA, https://www.cma.gov.cn/).

Bulk soils were collected from healthy steppe sites within the CAAS‐IGR Shaerqin Research Station to a depth of 20 cm from four points along a 200 m transect. Large pieces of debris were removed and then homogenised by hand. Six litres of homogenised bulk soil were then used to fill each mesocosm to a bulk density of ~1.25 g cm^−3^. For each mesocosm, six equally spaced sowing points were created and divided equally amongst the plant species required (Figure 1). Five surface‐sterilised seeds of the target plant species were sown at each sowing point. For mesocosms with two or more plant species, we ensured that adjacent sowing points contained different species to promote plant–plant interactions amongst different species. Seeds of all six plant species germinated ~8 days after sowing, and all sowing points were thinned to a seedling of a similar size.

All Phase 1 mesocosms were well‐watered (~60% soil moisture capacity) for the first 20 days to provide seedlings with sufficient soil moisture to establish in mesocosms before commencing the rainfall treatments (Figure 1). Mesocosms were then subjected to the respective rainfall treatments for ~3 months (i.e., 88 days; 20 June to 15 September 2020) with an automatic drip‐irrigation system (precision = 0.01 mL). We supplied either 40 mm (ambient rainfall amount) or 20 mm (50% reduction in total rainfall amount) of water over every 10‐day period for the two total rainfall amount treatments (at two frequencies) (Figure 1). The rainfall frequency treatments were set to every 5 (ambient) or 10 (50% reduction in frequency) days between two watering (i.e., ‘rainfall’) events (Figure 1). The ambient rainfall amount and frequency treatments were based on the monthly means of the growing seasons (June–September) between 1980 and 2018 (396 mm mean annual rainfall), the 50% reduction of total rainfall amount treatments was approximated based on the driest year on record of the study area (187 mm total rainfall in 2011), whilst the 50% reduction in rainfall frequency treatments involved lengthening the intervals between rainfall/watering events to twice the typical duration (CMA, https://www.cma.gov.cn/). Each treatment combination was replicated four times. After ~3 months (88 days), we harvested plants and recovered soil from each mesocosm. Soil recovered from each mesocosm was homogenised and stored in a cool (~4°C), dark room prior to the start of Phase 2.

### 2.5 Phase 2: Feedback Phase and Associated Measurements

For Phase 2, we sowed and grew the same number of plants and assemblages corresponding to the respective treatment combination from Phase 1 in smaller mesocosms within a greenhouse at the CAAS‐IGR Shaerqin Research Station (Figure 1). Within each Phase 2 mesocosm, we inoculated sterilised bulk soil with non‐sterilised soil (7% v/v) collected from the Phase 1 mesocosm previously containing the same plant species. We used bulk soil to minimise differences in soil abiotic properties amongst treatments, and bulk soil used in all Phase 2 mesocosms was sterilised with an autoclave for 2 h at 121°C and 15 psi. Additionally, we grew each of the 15 plant assemblage treatments in sterilised bulk soil alone to calculate biotic PSF values (Brinkman et al. 2010).

Each treatment combination was replicated four times. All plants were grown for 8 weeks (so that the mesocosms did not have time to become pot‐bound), maintained at ~20°C and ~60% soil moisture capacity. At the end of Phase 2, plants were harvested with shoots and roots of each species carefully separated, and then oven‐dried at 70°C for 72 h before determining dry biomass.

### 2.6 Statistical Analyses

We used log‐response ratios (LRRs) to calculate plant assemblage‐level biotic PSF effects (hereafter also referred to as ‘feedback effects’ for brevity) (Brinkman et al. 2010). The feedback effect on each Phase 2 plant assemblage, grown in soil conditioned by the same assemblage during Phase 1 under the respective rainfall amounts and frequencies, was calculated as:

**Feedback effect = log**_**10**_ **(Total plant biomass in mesocosm with non‐sterilised soil inoculum treatment ÷ Average total plant biomass of mesocosms with same assemblage in sterilised soil)**.

Feedback values are calculated as the relative growth responses of all plants in a mesocosm inoculated with non‐sterilised soil previously conditioned by the same species (i.e., assemblage) and subjected to specific total rainfall amounts and frequencies during Phase 1, compared to the average total plant growth in mesocosms containing the same assemblage with fully sterilised bulk soil (Brinkman et al. 2010; Kardol et al. 2007). The use of logarithmic transformation of biomass‐ratio values (i.e., LRR) to calculate feedback effect sizes enables simple relative comparisons of plant growth at mesocosm‐level amongst mesocosms containing various levels of plant functional group(s) or species with different net growth rates and growth forms. Next, the use of LRR enables comparisons of negative and positive feedback values proportional to their original effect sizes (Brinkman et al. 2010). Further, we used log_10_(*x*) to obtain LRRs for our study as a unit increase indicates that net plant growth of a given assemblage in non‐sterilised treatment soil is 10 times greater than that of growth in fully sterilised soil which, in turn, allows straightforward interpretation of PSF values.

Generalized least squares (GLS) models were used to compare the differences in feedback responses amongst different plant assemblages grown with soil inoculum previously subjected to contrasting total rainfall amount (per 10‐day period) and frequency treatments, and the interaction amongst these three factors. Plant assemblages, total rainfall amounts, and rainfall frequencies were treated as the main factors. The direction of each feedback value, whether significantly positive or negative (i.e., not zero), was determined by fitting the respective model showing significant interaction without intercept terms.

To investigate how the number of plant species or functional types moderates feedback effects during droughts (i.e., reduced rainfall amount and/or frequency), we calculated the absolute (or magnitude) difference in feedback effects (|Δ_feedback effect_|; modified from Veresoglou et al. 2022) between the specific reduced rainfall frequency and/or amount treatment versus the ambient rainfall treatment (i.e., 20 mm rainfall per 5‐day intervals) of each plant assemblage as:

**|Δ**_**feedback effect**_ **| = | (Feedback effect of mesocosm under specific reduced rainfall frequency and/or amount) – (Average feedback effect of mesocosms with same plant assemblage under ambient rainfall) |**.

A greater |Δ_feedback effect_| value indicates that the respective plant assemblage treatment had a greater difference in PSF effects between the specific reduced rainfall frequency and/or amount treatment versus the ambient rainfall treatment, whereas a smaller |Δ_feedback effect_| suggests PSF effects were more similar. Regression analyses with linear mixed‐effects (LME) models were then used to investigate the relationships between |Δ_feedback effect_| values of each reduced rainfall frequency and/or amount treatment and the number of plant species or functional types, with the type of plant assemblage treated as the random factor.

In addition, to better explore how the growth responses of each constituent plant species contributed to the overall feedback effect of each multi‐species mesocosm, we calculated the biotic effect of soil conditioning by each multi‐species plant assemblage under different rainfall treatments on constituent plant species growth in Phase 2 as:

**Biotic effect on constituent species biomass = Total biomass of a given species in mesocosm with multiple species and specific inoculated soil treatment – Average total biomass of conspecifics grown in mesocosms with same assemblage and sterilised soil**.

For each plant assemblage treatment containing multiple species in Phase 2, an LME model was used to compare the differences in total plant biomass accumulated in inoculated versus sterilised soil amongst the constituent plant species, the rainfall amounts and frequencies, and the interaction amongst these three factors; plant species, total rainfall amounts and rainfall frequencies were treated as main factors, with blocking by mesocosm replicate treated as a random factor.

Standardised residuals of every GLS or LME model were visually inspected to verify model assumptions, and Akaike Information Criterion and likelihood‐ratio tests were used to identify the appropriate model and variance structures for each comparison (Zuur et al. 2009). Post hoc Tukey's HSD (Honestly Significant Difference) tests were also conducted for each model when a main or interaction term of interest was significant (Hothorn et al. 2008). All statistical analyses were performed in ‘R’ (R Core Team 2022) using ‘nlme’ (Pinheiro et al. 2022), ‘multcomp’ (Hothorn et al. 2022), ‘MuMIn’ (Bartoń 2022) and ‘emmeans’ (Lenth et al. 2022) packages.

## 3 Results

### 3.1 PSF Shifts as Dependent on Rainfall Regime

**FIGURE 2:**
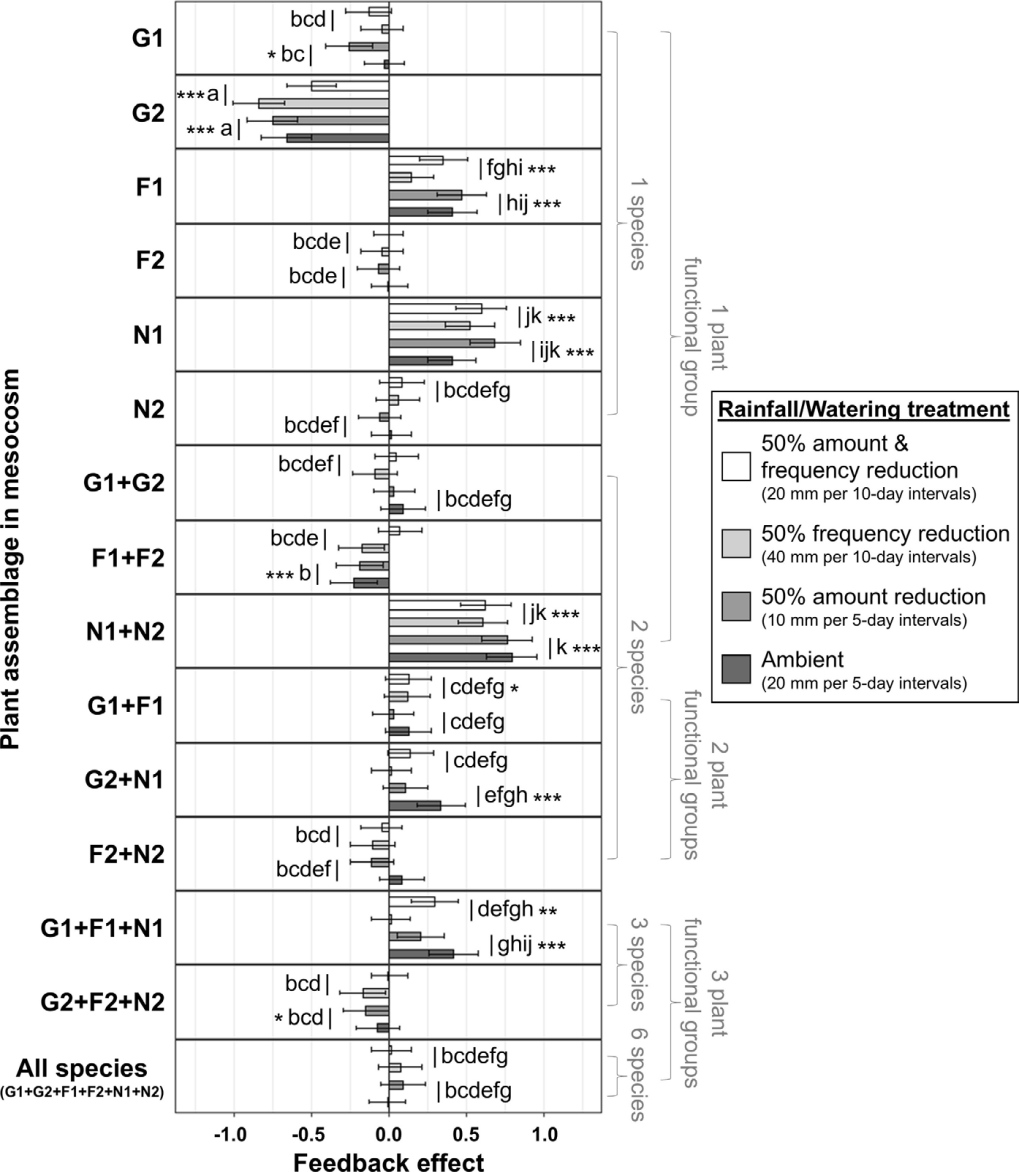
Biotic plant–soil feedback effect compared amongst 15 plant assemblages previously conditioned by the same plant assemblage subjected to four rainfall frequency and amount treatments (Figure 1). Feedback of each mesocosm treatment combination was calculated as log_10_(total biomass of plants grown in sterile bulk soil inoculated with 7% (v/v) unsterilised treatment soil ÷ average biomass of same plant assemblage grown in fully sterilised bulk soil). Bars are means and error bars represent 95% confidence intervals from a generalized least squares model (*n* = 4; Table S1). Within the significant ‘assemblage’ × ‘rainfall frequency’ interaction, different letters indicate significantly different means (post hoc Tukey's HSD test, *p* < 0.05), and *, **, *** indicate significant feedback value (i.e., direction) at *p* < 0.05, 0.01, or 0.001 (Table S2). G1, *Leymus chinensis* (grass); G2, *Stipa grandis* (grass); F1, *Allium ramosum* (forb); F2, *Artemisia sieversiana* (forb); N1, *Lespedeza daurica* (nitrogen‐fixing legume); N2, *Medicago ruthenica* (nitrogen‐fixing legume).

There was a significant effect of plant assemblage treatments on the magnitude and direction of feedback, whilst the factors of rainfall amount and frequency did not influence feedback effects when considered independently (Table S1). The effect of plant assemblage treatments on the magnitude and direction of feedback effects depended on rainfall frequency but was independent of total rainfall amount (Figure 2, Tables S1 and S2). Across all examined plant assemblage treatments, feedback effects also showed significant dependency on the interaction between rainfall frequency and amount (Figure S1, Table S1). However, we did not consider the combined influence of rainfall frequency and amount on feedback effects for further interpretation (Figure S1, Table S1) because the directions and magnitude of feedback differed markedly amongst the 15 plant assemblages (Figure 2, Tables S1 and S2), indicating that plant species composition is an important determinant of feedback effects. Further, there was no three‐way interactive effect involving plant assemblages, rainfall amounts and frequencies (Table S1).

Feedback effects did not significantly vary with rainfall frequencies within each plant assemblage treatment for all 15 plant assemblages (Figure 2, Tables S1 and S2). Under ambient rainfall frequency treatment, five plant assemblages showed significant positive feedback effects (i.e., ‘F1’, ‘N1’, ‘N1 + N2’, ‘G2 + N1’, ‘G1 + F1 + N1’), four plant assemblages showed significant negative feedback effects (i.e., ‘G1’, ‘G2’, ‘F1 + F2’, ‘G2 + F2 + N2’) and six plant assemblages showed non‐significant feedback effects (i.e., ‘F2’, ‘N2’, ‘G1 + G2’, ‘G1 + F1’, ‘F2 + N2’, ‘All species’) (Figure 2, Tables S1 and S2). On the other hand, under reduced rainfall frequency, nine plant assemblages showed non‐significant feedback effects (i.e., ‘G1’, ‘F2’, ‘N2’, ‘G1 + G2’, ‘F1 + F2’, ‘G2 + N1’, ‘F2 + N2’, ‘G2 + F2 + N2’, ‘All species’), five assemblages showed significant positive feedback effects (i.e., ‘F1’, ‘N1’, ‘N1 + N2’, ‘G1 + F1’, ‘G1 + F1 + N1’) and one assemblage showed significant negative feedback effects (i.e., ‘G2’) (Figure 2, Tables S1 and S2).

### 3.2 Moderation of PSF Shifts as Dependent on Plant Species or Functional Group Diversity

Feedback effects between both rainfall frequencies were similar within each of the 15 plant assemblages examined (Figure 2, Tables S1 and S2). Additionally, linear mixed‐effects models indicated that the magnitude of difference in feedback effects was unrelated to the number of plant species or functional group(s) when exposed to either of the three reduced rainfall frequency and/or amount treatments compared to the ambient rainfall amount and frequency treatment (Figure S2, Table S3).

### 3.3 Generalisation of Feedback Effects by Plant Functional Groups or Species

For each of the three plant functional groups, the magnitude and/or direction of feedback effects generally differed between the two species when grown in monocultures (Figure 2, Tables S1 and S2). For grasses, mesocosms containing only *S. grandis* (G2) showed significant negative feedback effects, and these effects were significantly more negative than those containing only *L. chinensis* (G1) (Figure 2, Tables S1 and S2). In addition, *L. chinensis* (G1) showed no feedback effects when subjected to reduced rainfall frequency, but showed significant negative feedback effects when subjected to the ambient rainfall frequency treatment (Figure 2; Tables S1 and S2). For forbs, mesocosms containing only *A. ramosum* (F1) generally showed significant positive feedback effects and were significantly more positive than those containing only *A. sieversiana* (F2), which showed no significant feedback effects under both rainfall frequency treatments (Figure 2, Tables S1 and S2). For the N‐fixing legumes, mesocosms containing only *L. daurica* (N1) showed significant positive feedback effects, and these effects were significantly more positive than those containing only *M. ruthenica* (N2), which showed no significant feedback under both rainfall frequency treatments (Figure 2, Tables S1 and S2).

**FIGURE 3:**
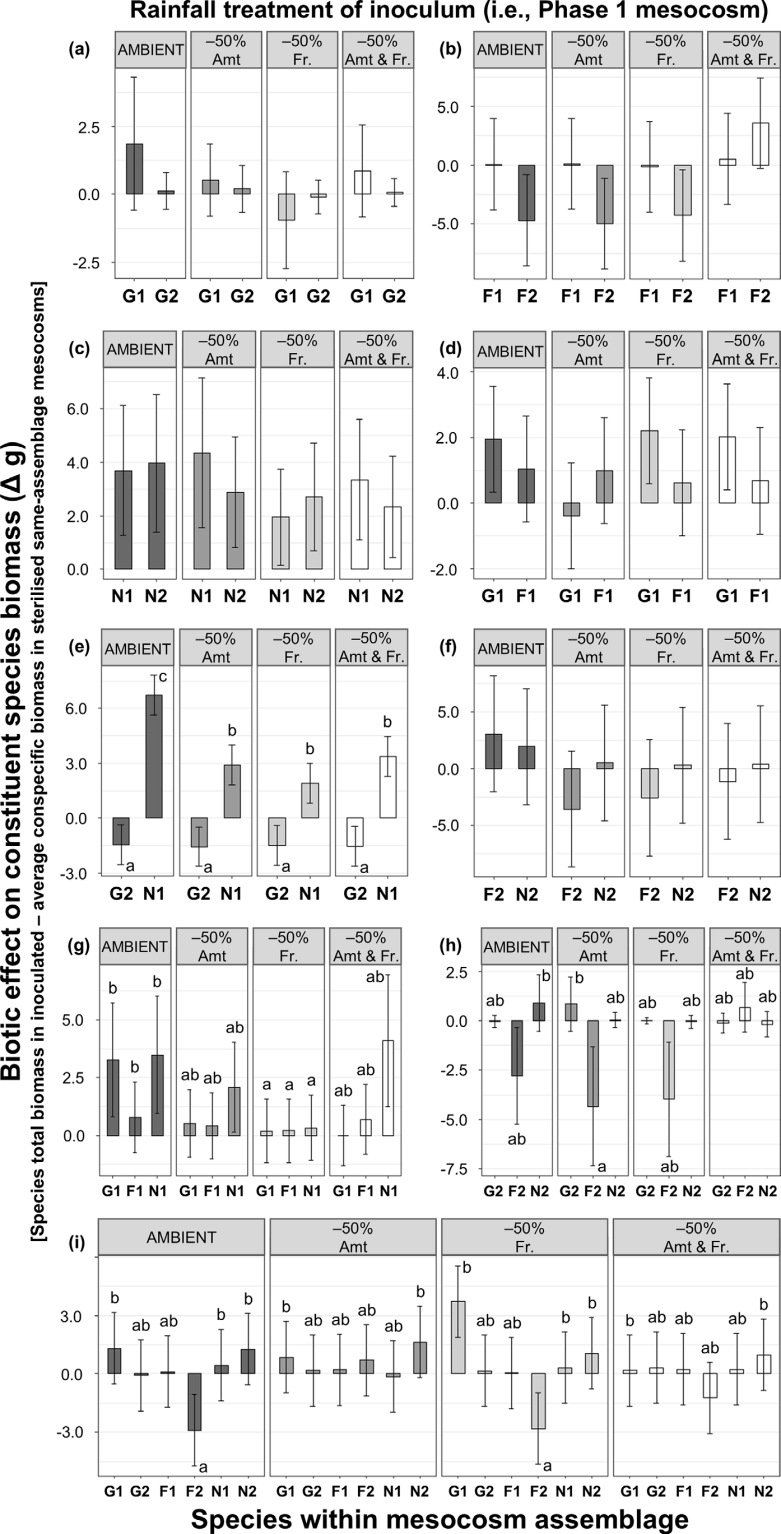
Biotic effect of Phase 1 soil conditioning by each multi‐species plant assemblage (represented by alphabetical panels; Figure 1) under different rainfall treatments on constituent plant species growth during Phase 2. For each assemblage, soil inocula utilised were conditioned by the same plant assemblage under different total rainfall amounts [Amt; 40 mm (ambient) or 20 mm (−50%) per 10‐day period] and frequencies [Fr.; 5‐day (ambient) or 10‐day intervals (−50%)] in Phase 1. Bars are means and error bars represent 95% confidence intervals from a linear mixed effects model within each panel (*n* = 4; Table S4). Different letters indicate significantly different means within each panel (post hoc Tukey's HSD test, *p* < 0.05). G1, *Leymus chinensis* (grass); G2, *Stipa grandis* (grass); F1, *Allium ramosum* (forb); F2, *Artemisia sieversiana* (forb); N1, *Lespedeza daurica* (nitrogen‐fixing legume); N2, *Medicago ruthenica* (nitrogen‐fixing legume).

In several cases, the direction of biomass responses of plant species grown in assemblage treatments containing heterospecific plants differed from the direction of feedback of the respective species grown in monocultures (Figures 2 and 3, Tables S1, S2 and S4). For example, monocultures of *S. grandis* (G2) (negative feedback), *A. ramosum* (F1) (positive feedback) and *L. daurica* (N1) (positive feedback) showed significant feedback across both rainfall frequency treatments (Figure 2, Tables S1 and S2), but each of these species showed negligible differences in total plant biomass between inoculated and sterilised treatments when grown together with heterospecific plants in certain assemblage treatments (e.g., G2 in ‘G1 + G2’ and ‘G2 + F2 + N2’ mesocosms, F1 in ‘F1 + F2’ mesocosms, and these three species in mesocosms containing all six plant species; Figure 3a,b,h,i, Table S4).

## 4 Discussion

Our study showed that whilst PSF effects varied amongst certain plant assemblages of the semi‐arid eastern Eurasian Steppe, these effects were similar within each assemblage type across different rainfall regimes. This result does not support our first hypothesis that PSF can be modified by rainfall regimes, nor our second hypothesis that increasing plant species richness or functional group diversity would moderate shifts in PSF with reduced rainfall amount and/or frequency. The lack of support for our second hypothesis was further demonstrated by the absence of a relationship between plant species richness or functional group diversity and PSF magnitude difference under ambient rainfall treatment versus treatments with reduced rainfall amount and/or frequency. Together, our results demonstrate that the resistance of plant–soil microbial community interactions to changing rainfall regimes in this semi‐arid steppe ecosystem might play a more important role in maintaining PSF stability than plant species richness or functional group diversity.

### 4.1 Influence of Rainfall Regimes on PSF

Our finding that the direction and magnitude of feedback effects were not significantly affected by changes in the amount and frequency of rainfall for all respective plant assemblage treatments suggests that PSF in this system are generally resistant to a growing season drought. This observation did not support our first hypothesis that PSF can be dependent on rainfall, but is in line with the findings of a meta‐analysis of drought‐related PSF studies showing that feedback effects of native plants often remain unchanged under both ambient and drought conditions (see Hassan et al. 2022). We speculate that the unchanged PSF response to the imposed reductions in rainfall amount and/or frequency is due to co‐evolved plant and soil microbial communities in this semi‐arid ecosystem, making the system well‐adapted to low water availability (de Vries et al. 2023; Evans and Wallenstein 2012; Lau and Lennon 2012; Schwinning and Ehleringer 2001; Soussi et al. 2016). Moreover, average monthly precipitation amounts outside of the examined growing season are comparable to or lower than our imposed drought treatments (e.g., ~19.1 mm per month in spring [1980–2018; CMA, https://www.cma.gov.cn/]—the driest season of the study area, Gao et al. 2014), which further suggests that outcomes of plant–soil interaction mechanisms, particularly biotic PSF, in this semi‐arid grassland are highly stable across a broad range of naturally occurring soil moisture conditions. Despite the negative impact of low water availability on plant growth performance and the microbial community imposed by the rainfall reduction treatments, the stability of biotic PSF (i.e., soil legacy) effects may be attributable to factors such as the presence of many microbial taxa within the community adapted to low water availability ensuring functional redundancy, resource‐efficient microbial traits that allow persistence on relatively low levels of plant‐derived substrates and nutrients, or the ability of many microbial taxa to go dormant under water‐stressed conditions (de Vries et al. 2023; Lau and Lennon 2012; Lennon and Jones 2011). However, our study did not examine soil microbial communities and their functional characteristics, and we recommend further research to unravel underlying mechanisms that promote stable plant community interactions in this ecosystem. Such information could inform the restoration of degraded semi‐arid grasslands, which are widespread on the eastern Eurasian Steppe, to help them better resist climatic extremes.

Nevertheless, it is important to note that our study used seedlings and explored only biotic PSF, which may not fully represent PSF in natural settings. This is because seedlings may exhibit heightened sensitivity to soilborne pathogens (Develey‐Rivière and Galiana 2007; Whalen 2005) or benefit from maternal investment in seed resources (De Long et al. 2023; de Vries et al. 2023; Vivas et al. 2020), potentially offsetting the effects of contrasting environments and associated microbial communities on other life history stages of the plant species. Also, our study did not examine abiotic PSF (e.g., alterations in soil nutrient availability or structure), which could also be influenced by rainfall regimes and, in turn, influence plant responses (Bennett and Klironomos 2019; Smith‐Ramesh and Reynolds 2017). Therefore, future studies should incorporate older plants and consider both biotic and abiotic PSF to better predict plant responses to changes in rainfall regimes under natural settings.

### 4.2 Moderation of PSF by Plant Diversity

Our second hypothesis was that increased plant diversity moderates the effects of drought on biotic PSF, but our results did not support this expectation. All plant assemblage treatments, including monocultures, did not show significant shifts in feedback effects between ambient versus reduced rainfall treatments. In addition, our second hypothesis was unsupported by the lack of a relationship between plant diversity and the feedback magnitude difference between ambient versus other rainfall regimes with reduced amount and/or frequency. This could be because interaction mechanisms amongst the examined plants and soil microorganisms of this semi‐arid environment are well‐adapted, and hence more resistant to drought as mentioned above (de Vries et al. 2023; Evans and Wallenstein 2012; Lau and Lennon 2012; Schwinning and Ehleringer 2001; Soussi et al. 2016).

Whilst to the best of our knowledge, no directly comparable PSF studies involving both plant diversity and drought factors currently exist, previous research on how plant diversity influences the responses of ecosystem functions to drought has yielded mixed results. Studies in several ecosystems have shown the beneficial effects of plant diversity on the resistance of ecosystem functions to drought, and ascribe these effects to mechanisms such as resource use complementarity or facilitation (e.g., see Blondeel et al. 2024; Gazol and Camarero 2016; Gillespie et al. 2020; Grossiord et al. 2014; Li et al. 2022; Lüscher et al. 2022; Ploughe et al. 2019). However, other studies have shown no significant effects of plant diversity on drought resistance or have instead demonstrated the importance of species‐specific traits (e.g., drought‐tolerant species disproportionately driving community‐level responses) (e.g., see Elsalahy et al. 2020; Grossiord et al. 2014; Komainda et al. 2020; Lüscher et al. 2022; Paligi et al. 2024; Ploughe et al. 2019). The varied findings amongst previous studies suggest that mechanisms by which diversity influences ecosystem functioning during drought may be ecosystem‐dependent and difficult to generalise (e.g., Grossiord et al. 2014). In our semi‐arid ecosystem, we found that plant species richness and functional group diversity had limited influence on the stability of biotic PSF across the examined rainfall regimes, highlighting the need to consider drought resistance in species‐specific interactions in mediating PSF, rather than a single focus on maximising plant diversity when restoring and managing ecosystems.

### 4.3 Feedback Effects by Plant Functional Groups or Species

PSF effects detected in this study are difficult to generalise based on the responses of broad plant functional groups under various rainfall treatments. First, the direction and/or magnitude of PSF responses varied significantly between plant species within each functional group, which is consistent with previous work showing that PSF effects are difficult to predict using broad plant functional groups of graminoids and forbs (Spitzer et al. 2022). Second, the direction of feedback of each plant species growing in monocultures was generally not indicative of growth responses of the respective species grown in multi‐species mixtures, which suggests that competitive or facilitative interactions amongst different plant species may alter feedback effects differently from those observed in monocultures (Hendriks et al. 2013; Wubs and Bezemer 2016). Together, these observations support the view that future PSF studies should simultaneously consider a greater number of factors or traits (e.g., competition, facilitation, root and/or hydraulic traits) to better determine the strength and direction of PSF (e.g., De Long et al. 2023; Gundale and Kardol 2021; Png et al. 2023) under altered rainfall regimes, which can help project the impacts of climate change on vegetation composition.

## 5 Conclusions

We showed that, within each plant assemblage type, the PSF of all examined plant assemblages in this semi‐arid steppe ecosystem were unaffected by drier rainfall regimes during the growing season. Furthermore, higher plant diversity (i.e., species and functional groups) did not lessen the magnitude differences in PSF between ambient and reduced rainfall treatments. The limited influence of drought on PSF effects across the examined plant assemblages might be because many plants and co‐evolved microorganisms of this semi‐arid environment are well‐adapted to the imposed rainfall reduction regimes. Overall, our study highlights that plant diversity alone does not moderate PSF effects across varying rainfall regimes, underscoring the complexity of the underlying mechanisms and the need to consider additional plant or microbial traits (e.g., drought tolerance) and their interactive responses, which will allow us to better predict and mitigate drought impacts on PSF and their consequences on plant community dynamics.

## Author Contributions

**Xiliang Li:** data curation, formal analysis, funding acquisition, methodology, project administration, resources, supervision, writing – original draft, writing – review and editing. **G. Kenny Png:** conceptualization, data curation, formal analysis, methodology, supervision, writing – original draft, writing – review and editing. **Zhen Zhang:** methodology, writing – review and editing. **Fenghui Guo:** methodology, writing – review and editing. **Yuanheng Li:** methodology, writing – review and editing. **Fang Li:** methodology, writing – review and editing. **Shan Luo:** methodology, writing – review and editing. **Nicholas J. Ostle:** funding acquisition, methodology, project administration, supervision, writing – review and editing. **John N. Quinton:** funding acquisition, supervision, writing – review and editing. **Urs A. Schaffner:** funding acquisition, methodology, supervision, writing – review and editing. **Xiangyang Hou:** funding acquisition, project administration, supervision, writing – review and editing. **David A. Wardle:** methodology, supervision, writing – original draft, writing – review and editing. **Richard D. Bardgett:** conceptualization, funding acquisition, methodology, project administration, supervision, writing – original draft, writing – review and editing.

## Conflicts of Interest

The authors declare no conflicts of interest.

## Supporting information


Data S1.

## Data Availability

Data available from the Dryad Digital Repository: https://doi.org/10.5061/dryad.7d7wm385s (Li et al. 2025).
