# Supplementary material for: Higher Plant Diversity Does Not Moderate the Influence of Changing Rainfall Regimes on Plant–Soil Feedback of a Semi‐Arid Grassland
Source: Glob Chang Biol. 2025 Mar 4;31(3):e70084. doi: 10.1111/gcb.70084 (PMC11877630; doi:10.1111/gcb.70084)
Supplement: Supplementary file 1 — Data S1. [file GCB-31-e70084-s001.pdf]

SUPPORTING INFORMATION: Higher Plant Diversity Does Not Moderate the Influence of Changing Rainfall Regimes on Plant-Soil Feedback of a Semi-Arid Grassland

Xiliang Li[1†] | G. Kenny Png[2†] | Zhen Zhang[1] | Fenghui Guo[1,3] | Yuanheng Li[1] | Fang Li[1] | Shan Luo[4,5] | Nicholas J. Ostle[4] | John N. Quinton[4] | Urs A. Schaffner[6] | Xiangyang Hou[1,7] | David A. Wardle[8] | Richard D. Bardgett[2,4]

[1]Institute of Grassland Research, Chinese Academy of Agricultural Sciences, Hohhot, China | [2]Department of Earth and Environmental Sciences, The University of Manchester, Manchester, UK | [3]The Industrial Crop Institute, Shanxi Agriculture University, Taiyuan, China | [4]Lancaster Environment Centre, Lancaster University, Lancaster, UK | [5]Department of Evolution, Ecology and Behaviour, University of Liverpool, Liverpool, UK | [6]Centre for Agriculture and Biosciences International, Delémont, Switzerland | [7]College of Grassland Science, Shanxi Agriculture University, Taigu, China | [8]Department of Ecology and Environmental Science, Umeå Universitet, Umeå, Sweden

†Both authors contributed equally to this work and should be considered joint first authors

Correspondence: G. Kenny Png (gcpng.kenny@gmail.com); Fang Li (lifang_418007@163.com)

SI Tables

TABLE S1 | Results of three-factor generalized least squares (GLS) model fitted with intercept terms to test for the effects of plant assemblage type (PA; see Figure 1 for treatment details), total rainfall amount (RA), rainfall frequency (RF), and their interactions on plant-soil feedback effects. Significant *p*-values ($p < 0.05$) are presented in bold

Source of variation	GLS test statistics		
	d.f. $_{(n, d)}$	*F*	*p*
Factor(s) of model fitted with intercept terms			
Plant assemblage (PA)	14, 180	61.22	**< 0.001**
Total rainfall amount over 10 days (RA)	1, 180	1.78	0.184
Rainfall frequency (RF)	1, 180	1.16	0.283
PA × RA	14, 180	1.53	0.105
PA × RF	14, 180	2.01	**0.019**
RA × RF	1, 180	18.43	**< 0.001**
PA × RA × RF	14, 180	1.58	0.089

TABLE S2 | Results of three-factor generalized least squares (GLS) model fitted without intercept terms to test for the significance of each plant-soil feedback effect value (i.e., significantly different from zero) for each treatment combination of plant assemblage type (see Figure 1 for treatment details) × rainfall frequency (RF). Main factors of the three-factor GLS model are plant assemblage type, total rainfall amount and rainfall frequency. Significant *p*-values ($p < 0.05$) are presented in bold

Source of variation	GLS test statistics		
	d.f. $_{(n, d)}$	*t*	*p*
Groups of model fitted without intercept terms			
‘G1’ × ‘50% reduction’ rainfall frequency (RF)	1, 240	-1.62	0.107
‘G1’ × ‘ambient’ RF	1, 240	-2.53	**0.012**
‘G2’ × ‘50% reduction’ RF	1, 240	-10.04	**< 0.001**
‘G2’ × ‘ambient’ RF	1, 240	-10.48	**< 0.001**
‘F1’ × ‘50% reduction’ RF	1, 240	4.19	**< 0.001**
‘F1’ × ‘ambient’ RF	1, 240	6.92	**< 0.001**
‘F2’ × ‘50% reduction’ RF	1, 240	-0.46	0.649
‘F2’ × ‘ambient’ RF	1, 240	-0.64	0.526
‘N1’ × ‘50% reduction’ RF	1, 240	8.59	**< 0.001**
‘N1’ × ‘ambient’ RF	1, 240	8.39	**< 0.001**
‘N2’ × ‘50% reduction’ RF	1, 240	1.38	0.168
‘N2’ × ‘ambient’ RF	1, 240	-0.43	0.670
‘G1+G2’ × ‘50% reduction’ RF	1, 240	-0.41	0.682
‘G1+G2’ × ‘ambient’ RF	1, 240	1.24	0.216
‘F1+F2’ × ‘50% reduction’ RF	1, 240	-0.99	0.321
‘F1+F2’ × ‘ambient’ RF	1, 240	-3.52	**< 0.001**
‘N1+N2’ × ‘50% reduction’ RF	1, 240	9.33	**< 0.001**
‘N1+N2’ × ‘ambient’ RF	1, 240	11.50	**< 0.001**
‘G1+F1’ × ‘50% reduction’ RF	1, 240	2.24	**0.026**
‘G1+F1’ × ‘ambient’ RF	1, 240	1.49	0.137
‘G2+N1’ × ‘50% reduction’ RF	1, 240	1.53	0.127
‘G2+N1’ × ‘ambient’ RF	1, 240	3.79	**< 0.001**
‘F2+N2’ × ‘50% reduction’ RF	1, 240	-1.44	0.152
‘F2+N2’ × ‘ambient’ RF	1, 240	-0.26	0.794
‘G1+F1+N1’ × ‘50% reduction’ RF	1, 240	2.75	**0.007**
‘G1+F1+N1’ × ‘ambient’ RF	1, 240	5.12	**< 0.001**
‘G2+F2+N2’ × ‘50% reduction’ RF	1, 240	-1.52	0.129
‘G2+F2+N2’ × ‘ambient’ RF	1, 240	-2.00	**0.047**
‘All species’ × ‘50% reduction’ RF	1, 240	0.94	0.346
‘All species’ × ‘ambient’ RF	1, 240	0.89	0.376

TABLE S3 | Regression analyses of one-factor linear mixed-effects (LME) models to test for the effects of (i) the number of plant functional groups (NPFG) or (ii) species (NPSP) on the absolute difference in plant-soil feedback magnitude of three respective rainfall combination treatments (|Δfeedback effect|). Plant assemblage type was treated as the random factor in the LME model

Source of variation	LME test statistics				
Rainfall combination treatment	$R^2_{\text{conditional}}$	R^2_{marginal}	d.f. $_{(n, d)}$	*F*	*p*
50% amount reduction (10 mm rain per 5-day intervals)					
a) Number of plant functional groups (NPFG)	0.198	0.002	1, 13	0.081	0.780
b) Number of plant species (NPSP)	0.197	0.012	1, 13	0.474	0.503
50% frequency reduction (40 mm rain per 10-day intervals)					
c) NPFG	0.261	0.020	1, 13	0.695	0.419
d) NPSP	0.263	0.001	1, 13	0.036	0.852
50% amount & frequency reduction (20 mm rain per 10-day intervals)					
e) NPFG	0.004	< 0.001	1, 13	0.011	0.918
f) NPSP	0.004	< 0.001	1, 13	0.032	0.860

TABLE S4 | Results of three-factor linear mixed-effects (LME) models fitted with intercept terms to compare the effects of constituent plant species within each assemblage type (SP; see Figure 1 for treatment details), total rainfall amount over 10 days (RA), rainfall frequency (RF), and their interactions on the difference in total biomass of constituent plant species in mesocosms inoculated with unsterilised soil versus the average total biomass of the same species grown in mesocosms with sterilised soil containing the same plant assemblage. Mesocosm replicate number was treated as the random factor in the LME model. Significant *p*-values ($p < 0.05$) are presented in bold

Source of variation	LME test statistics		
	d.f. $_{(n, d)}$	*F*	*p*
<u>Factor(s) of model fitted with intercept terms</u>			
(a) G1 + G2			
Plant species (SP)	1, 21	0.733	0.401
Total rainfall amount over 10 days (RA)	1, 21	0.322	0.577
Rainfall frequency (RF)	1, 21	0.830	0.373
SP × RA	1, 21	0.188	0.669
SP × RF	1, 21	0.335	0.569
RA × RF	1, 21	0.533	0.473
SP × RA × RF	1, 21	2.573	0.124
(b) F1 + F2			
SP	1, 21	4.304	0.051
RA	1, 21	2.431	0.134
RF	1, 21	2.994	0.098
SP × RA	1, 21	1.661	0.211
SP × RF	1, 21	2.749	0.112
RA × RF	1, 21	2.758	0.112
SP × RA × RF	1, 21	2.005	0.171
(c) N1 + N2			
SP	1, 21	0.004	0.951
RA	1, 21	0.248	0.624
RF	1, 21	3.524	0.075
SP × RA	1, 21	2.634	0.120
SP × RF	1, 21	0.307	0.586
RA × RF	1, 21	0.355	0.558
SP × RA × RF	1, 21	0.0001	0.991
(d) G1 + F1			
SP	1, 21	1.284	0.270
RA	1, 21	1.350	0.258
RF	1, 21	0.780	0.387
SP × RA	1, 21	1.384	0.253
SP × RF	1, 21	2.467	0.131
RA × RF	1, 21	1.106	0.305
SP × RA × RF	1, 21	0.883	0.358

(MORE)

Source of variation	LME test statistics		
	d.f. $_{(n, d)}$	*F*	*p*
Factor(s) of model fitted with intercept terms			
(e) G2 + N1			
SP	1, 21	197.096	**< 0.001**
RA	1, 21	2.916	0.103
RF	1, 21	8.608	**0.008**
SP × RA	1, 21	2.142	0.158
SP × RF	1, 21	8.301	**0.009**
RA × RF	1, 21	12.732	**0.002**
SP × RA × RF	1, 21	12.243	**0.002**
(f) F2 + N2			
SP	1, 21	1.094	0.308
RA	1, 21	0.864	0.363
RF	1, 21	0.495	0.490
SP × RA	1, 21	0.303	0.588
SP × RF	1, 21	0.042	0.840
RA × RF	1, 21	1.856	0.188
SP × RA × RF	1, 21	0.874	0.361
(g) G1 + F1 + N1			
SP	2, 33	2.314	0.115
RA	1, 33	0.089	0.767
RF	1, 33	3.079	0.089
SP × RA	2, 33	1.542	0.229
SP × RF	2, 33	0.701	0.503
RA × RF	1, 33	5.417	**0.026**
SP × RA × RF	2, 33	1.368	0.269
(h) G2 + F2 + N2			
SP	2, 33	1.882	0.168
RA	1, 33	0.204	0.654
RF	1, 33	0.0001	0.992
SP × RA	2, 33	4.346	**0.021**
SP × RF	2, 33	2.474	0.100
RA × RF	1, 33	0.029	0.865
SP × RA × RF	2, 33	4.349	**0.021**
(i) All species			
SP	5, 69	5.527	**< 0.001**
RA	1, 69	0.116	0.734
RF	1, 69	0.008	0.931
SP × RA	5, 69	2.555	**0.035**
SP × RF	5, 69	0.435	0.823
RA × RF	1, 69	1.235	0.270
SP × RA × RF	5, 69	0.579	0.716

TABLE S5 | Results of three-factor generalized least squares (GLS) model fitted without intercept terms to test for the significance of each plant-soil feedback effect value (i.e., significantly different from zero) for each treatment combination of total rainfall amount (RA) × rainfall frequency (RF). Main factors of the three-factor GLS model are plant assemblage (see Figure 1 for treatments), total rainfall amount and rainfall frequency. Significant *p*-values ($p < 0.05$) are presented in bold

Source of variation	GLS test statistics		
	d.f. $_{(n, d)}$	*t*	*p*
Groups of models fitted without intercept terms			
’50% reduction’ total rainfall amount (RA) × ‘50% reduction’ rainfall frequency (RF)	1, 240	2.567	**0.011**
’50% reduction’ RA × ‘ambient’ RF	1, 240	1.240	0.216
’Ambient’ RA × ‘50% reduction’ RF	1, 240	0.167	0.868
’Ambient’ RA × ‘ambient’ RF	1, 240	2.584	**0.010**

SI Figures

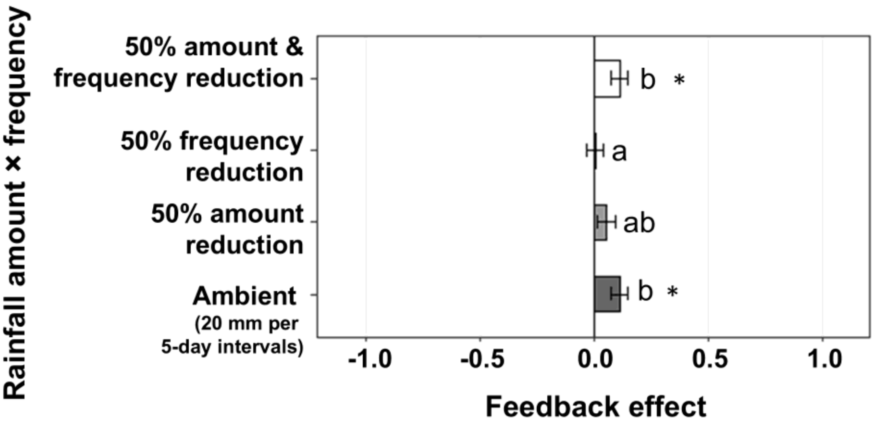

FIGURE S1 | Biotic plant-soil feedback effect compared across all 15 plant assemblages grown in soil previously conditioned by the same respective assemblage exposed to two different total amounts of rainfall [ambient = 40 mm, or 50% reduction from ambient = 20 mm per 10-day period] delivered at two frequencies [ambient = 5-day, or 50% reduction = 10-day intervals) (Figure 1). Feedback of each mesocosm treatment combination used for analyses was calculated as $\log_{10}$(total biomass of plants grown in sterile bulk soil inoculated with 7% (v/v) unsterilised treatment soil ÷ average biomass of same plant assemblage grown in fully sterilised bulk soil). Bars are means and error bars represent 95% confidence intervals from a generalized least squares model (Table S1). Different letters indicate significantly different means (post hoc Tukey's HSD test, $p < 0.05$), and *, **, *** indicate significant feedback value at $p < 0.05$, 0.01, or 0.001 (Table S5) for the 'rainfall total amount' × 'rainfall frequency' interaction.

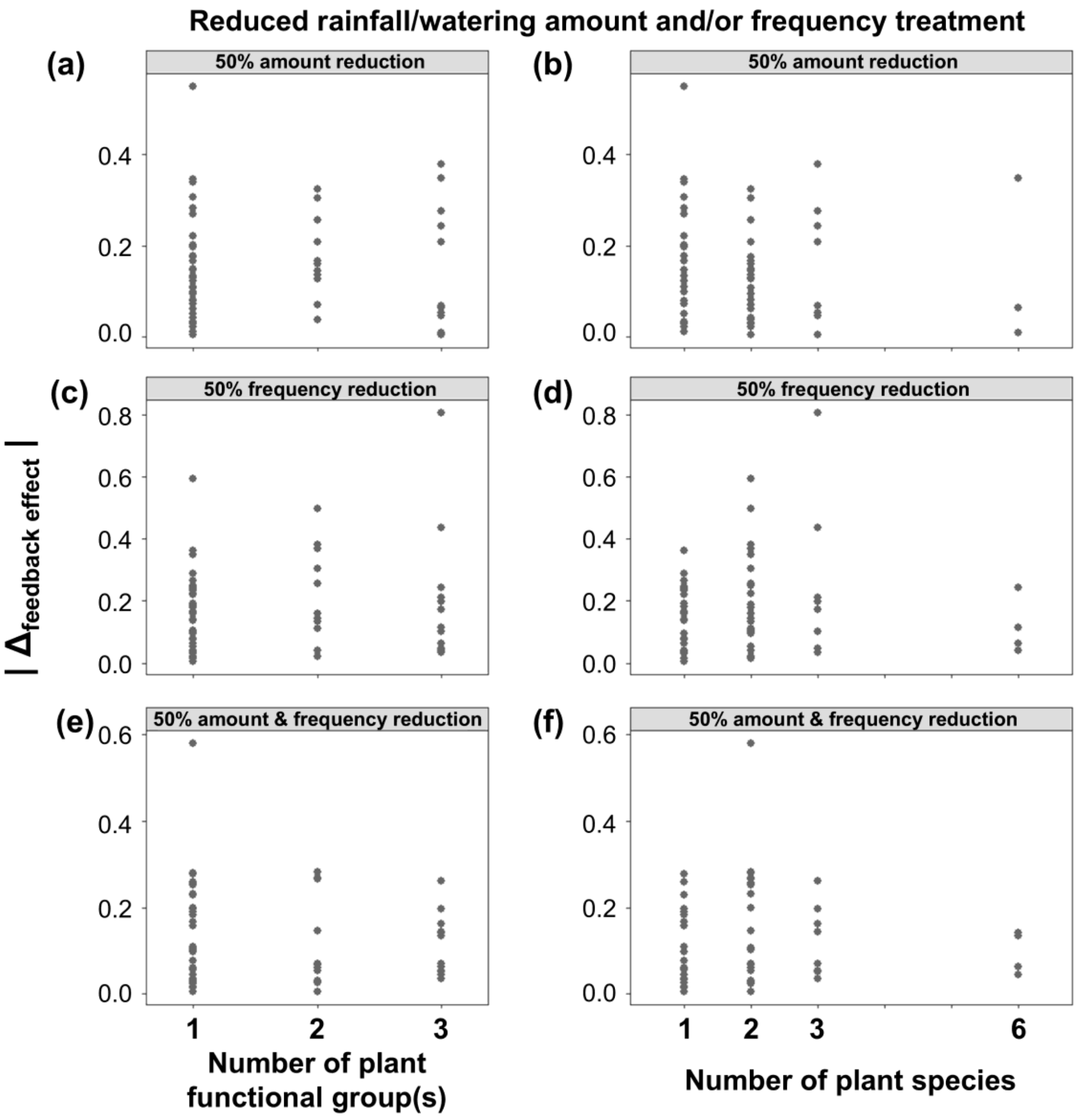

FIGURE S2 | Relationships between the number of plant (a, c, e) functional group(s) or (b, d, f) species and the absolute magnitude difference in biotic plant-soil feedback ($|\Delta_{\text{feedback effect}}|$) between a specific reduced rainfall frequency and/or amount treatment versus ambient rainfall treatment (i.e., 20 mm rainfall per 5-day intervals). $|\Delta_{\text{feedback effect}}|$ of each mesocosm was calculated as |'feedback effect in reduced rainfall frequency and/or amount treatment' – 'average feedback effect in ambient rainfall treatment containing same plant assemblage'|. No relationship was found for any panel based on linear mixed-effects models (i.e., all $p > 0.05$; Table S3).
